# Was kommt nach CDK4/6-Inhibition? Perspektiven beim fortgeschrittenen Mammakarzinom

**DOI:** 10.1007/s00508-025-02582-y

**Published:** 2025-07-31

**Authors:** Vanessa Castagnaviz, Anna Fenzl, Michael Gnant, Rupert Bartsch

**Affiliations:** 1 Department of Internal Medicine III with Hematology, Medical Oncology, Hemostaseology, Infectiology and Rheumatology, Oncologic Center, Salzburg Cancer Research Institute – Laboratory for Immunological and Molecular Cancer Research (SCRI-LIMCR), Paracelsus Medical University Salzburg, Salzburg, Österreich; 2 Springer-Verlag GmbH, Wien, Österreich; 3 Comprehensive Cancer Center, Austrian Breast and Colorectal Cancer Study Group, Medical University of Vienna, Waehringer Guertel 18–20, 1090 Wien, Österreich; 4 Department of Medicine I, Division of Oncology, Medical University of Vienna, Wien, Österreich; 5 Landeskrankenhaus Salzburg, Müllner Hauptstraße, 5020 Salzburg, Österreich

## Einleitung

Cyklin-abhängige Kinase-4/6-Inhibitoren (CDK4/6i) haben die Behandlung des HR+/HER2− (Hormonrezeptor-positiven, humaner epidermaler Wachstumsfaktor-Rezeptor-2-negativen) Mammakarzinoms revolutioniert und sind zum Standard in der ersten Linie der endokrinen Therapie (ET) geworden [1, 2, 3, 4, 5, 6, 7, 8]. Die aktuellen Leitlinien empfehlen den frühzeitigen Einsatz von CDK4/6i beim HR+ metastasierten Mammakarzinom (mBC) in der Mehrheit der Therapieszenarien [9, 10, 11]. In der klinischen Praxis besteht jedoch insbesondere bei Patientinnen mit Hochrisikokonstellationen – etwa bei hoher Tumorlast, symptomatischer, rasch progredienter Erkrankung oder einer lebensbedrohlichen viszeralen Krise, die eine schnelle Krankheitskontrolle erfordert – weiterhin Unsicherheit über das optimale therapeutische Vorgehen. Die Phase-IV-Studie PADMA [12] untermauert die Ergebnisse der Phase-II-Studie RIGHT Choice [13] und zeigt anhand praxisnaher Daten eine potenzielle Überlegenheit der endokrinbasierten Therapie mit Palbociclib gegenüber einer Monochemotherapie selbst in dieser prognostisch ungünstigen Subgruppe [12]. Gleichzeitig konnte die SONIA-Studie für den sofortigen Einsatz eines CDK4/6i in der Erstlinie keinen Vorteil im Gesamtüberleben gegenüber einer sequenziellen Anwendung belegen [14, 15], was den Bedarf an individualisierten Therapieentscheidungen zusätzlich unterstreicht. Bis dato gibt es drei zugelassene CDK4/6i bei mBC in Europa, die ein ähnliches progressionsfreie Überleben (PFS) gezeigt haben [1, 6, 16]. Die Erstlinienzulassung (1L) von Palbociclib basiert auf den Ergebnissen der Phase-III-Studie PALOMA‑2 (NCT01740427) [1], Ribociclib auf der Phase-III-Studie MONALEESA‑2 (NCT01958021) [16, 17] und Abemaciclib auf der Phase-III-Studie MONARCH 3 (NCT02246621) [6]. In Bezug auf das Gesamtüberleben (OS) zeigte nur Ribociclib in MONALEESA‑2 einen signifikanten Vorteil mit einem medianen OS von 63,9 Monaten versus 51,4 Monaten im Kontrollarm (HR 0,76; *p* = 0,008) [17]. Abemaciclib erzielte in MONARCH 3 ein medianes OS von 66,8 vs. 53,7 Monaten (HR 0,804; *p* = 0,066) [18]. Palbociclib erreichte in PALOMA‑2 kein signifikant verlängertes OS (53,9 vs. 49,8 Monate; HR 0,92; one-sided *p* = 0,21) [19]. Bis dato existieren nur begrenzte Daten zur optimalen therapeutischen Reihenfolge nach CDK4/6i, und obwohl zahlreiche Optionen verfügbar sind, bleibt die Evidenz, die die effektivste Sequenz unterstützt, spärlich. Dennoch wird eine „serielle ET“ gefolgt von zytotoxischen Wirkstoffen von allen aktuellen Behandlungsrichtlinien empfohlen.

Dieses Literaturstudium zielt darauf ab, aktuelle Informationen über mögliche Strategien nach dem Fortschreiten unter CDK4/6i bereitzustellen, indem verfügbare Daten zu Zweit- und Drittlinientherapieoptionen nach der Behandlung mit CDK4/6i beleuchtet werden.

## Was tun bei Progress? – Resistenzen

Während die CDK4/6i-basierte Therapie in der Erstlinie relevante klinische Wirksamkeit zeigt, weisen etwa 20 % der mit einer Kombination aus CDK4/6i und ET behandelten Tumoren eine primäre Resistenz (*de novo*) auf, die sich durch ein Ausbleiben des initialen Ansprechens oder einen nur begrenzt anhaltenden klinischen Nutzen auszeichnet. Darüber hinaus entwickeln nahezu alle Tumoren schließlich eine erworbene (sekundäre) Resistenz, was den Einsatz weiterer Therapielinien erforderlich macht [20, 21, 22].

**Abb. 1:**
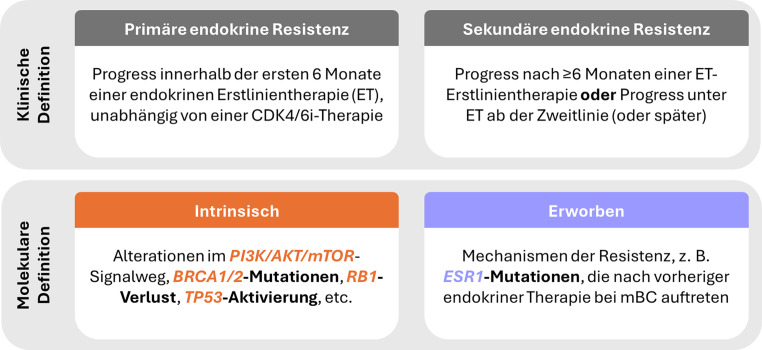
Resistenz gegenüber endokriner Therapie (*ET*) bei ER+/HER2− metastasiertem Mammakarzinom (*mBC*). Primäre endokrine Resistenz [22], sekundäre endokrine Resistenz [22], Intrinsisch [23, 24, 25], Erworben [23, 28]

Die Klassifikation der endokrinen Resistenz lässt sich aber nicht nur anhand klinischer, sondern auch molekularer Parameter vornehmen (siehe Abb. 1). Demnach fußt die intrinsische Resistenz z. B. auf aktivierenden Mutationen im PI3K/AKT/mTOR-Signalweg, *BRCA1/2*-Mutationen, *RB1*-Verlust oder *TP53*-Aktivierung [23, 24, 25], während die erworbene Resistenz häufig durch *ESR1*-Mutationen charakterisiert ist, welche typischerweise unter endokriner Therapie im metastasierten Stadium auftreten [23, 26]. Basierend auf diesen molekularbiologischen Faktoren sollte unter Berücksichtigung von Klinik und patientinnenspezifischen Faktoren die jeweils passendste Folgetherapielinie ausgewählt werden. Daten aus dem österreichischen Register für mBC der Arbeitsgemeinschaft medikamentöse Tumortherapie gemeinnützige GmbH (AGMT MBC-Register) zeigen Therapieabbruchsraten von 19,6 % zwischen der ersten und zweiten Therapielinie, aufgrund schlechten Allgemeinzustandes, einer Ablehnung weiterer Therapien oder weil die Patientinnen vor Beginn der nächsten Therapielinie verstarben [27]. Zusammen mit der verfügbaren wissenschaftlichen Evidenz unterstreicht dies die Bedeutung einer „Best-Treatment-First“-Strategie – wenngleich diese stets gegen potenzielle Toxizitäten unter Berücksichtigung der individuellen Präferenz abgewogen werden muss. Um möglichst vielen Patientinnen den Zugang zur wirksamsten Therapie zu ermöglichen, gilt es darüber hinaus zu verstehen, wie sich die Exposition gegenüber ET und CDK4/6i auf den Phänotyp und/oder das genomische Profil metastatischer Tumoren auswirkt – insbesondere im Hinblick auf potenziell therapierbare Mutationen.

Nach dem Eintreten einer Progression unter AI + CDK4/6i sollte zunächst eine molekulargenetische (Re‑)Testung auf HER2- und HR-Status, somatische Mutationen (Gewebe oder Liquid Biopsy) sowie Keimbahnmutationen wie *BRCA1/2* erfolgen. Eine Progression nach endokriner Vortherapie oder CDK4/6-Inhibition ist häufig mit dem Auftreten einer endokrinen Resistenz assoziiert, die unter anderem durch erworbene Mutationen in Genen wie *ESR1, ERBB2* oder *NF1* vermittelt wird. NGS-basierte ctDNA-Analysen ermöglichen dabei nicht nur die Identifikation therapeutisch relevanter Alterationen, sondern auch die dynamische Erfassung molekularer Veränderungen im Krankheitsverlauf [29]. Sofern eine therapierbare molekulare Alteration identifiziert wurde, ist eine Fortführung der ET in Kombination mit einem zielgerichteten Wirkstoff angezeigt – vorausgesetzt, es liegt keine endokrine Resistenz vor und die Patientin ist für diese Strategie geeignet (s. unten) ist hingegen kein ausreichendes Ansprechen auf die ET zu erwarten, sollten alternative Optionen wie Chemotherapie und Antikörper-Wirkstoffkonjugate erwogen werden.

## Anhaltende Bedeutung der Biomarker-Testung

Gewebebiopsien gelten weiterhin als diagnostischer Goldstandard zur Charakterisierung des Gewebes einschließlich der Feststellung des HR- und HER2-Status sowie der Identifikation genomischer Alterationen. Allerdings entwickeln Studien zufolge bis zu 40 % der Tumoren unter dem Druck der Therapie biologische Veränderungen mit potenzieller therapeutischer Relevanz [30, 31, 32, 33, 34, 35, 36, 37, 38]. Die Liquid Biopsy, insbesondere die Analyse zirkulierender Tumor-DNA (ctDNA), hat sich daher als ergänzendes Instrument zur herkömmlichen Gewebebiopsie etabliert [30, 34, 39, 40, 41].

Die AURORA-Studie identifizierte bei Patientinnen mit HR+ mBC häufige Mutationen in verschiedenen Treibergenen wie *ESR1, PTEN, PIK3CA* oder *RB1* sowie eine hohe Tumormutationslast (TMB), die mit einem erhöhten Rezidivrisiko assoziiert war [34, 42]. Besonders *ESR1*-Mutationen treten im metastasierten Setting zunehmend auf – unter Aromatasehemmer (AI)-Therapie steigen die Nachweisraten von < 1 % im Primärtumor auf etwa 5 % in der Erstlinie und schließlich auf 33 % bzw. ca. 40 % in der Zweit- bzw. Drittlinie [28, 43, 44, 45, 46]. Die PADA-1-Studie und kürzlich auch die SERENA‑6 Studie zeigten, dass der Wechsel von AI zu einem SERD (Fulvestrant bzw. Camizestrant) bei Nachweis einer *ESR1*-Mutation – unter Beibehaltung desselben CDK4/6i – das PFS gegenüber einer Fortführung der Aromatasehemmertherapie ab Randomisierung nahezu verdoppelte [47, 48]. Dies unterstreicht die potenzielle klinische Bedeutung der seriellen ctDNA-Testung zur frühzeitigen Detektion von Resistenzen und zur gezielten Therapieanpassung [49, 50, 51, 52] – allerdings müssen vor einer breiten Umsetzung in der klinischen Praxis noch weitere Studiendaten abgewartet werden, insbesondere zu einem möglichen Gesamtüberlebensvorteil, zur Wirksamkeit über Folgetherapien hinweg, zur praktischen Umsetzbarkeit im Versorgungsalltag sowie zur Kosten-Effektivität.

Obwohl auch Gewebe mittels Next-Generation Sequencing (NGS) analysiert werden kann, erfolgen serielle Gewebebiopsien aufgrund ihrer Invasivität und/oder anatomischen Einschränkungen (z. B. Knochenmetastasen) nicht routinemäßig [29]. Dies unterstreicht zusätzlich die Relevanz der plasmabasierten ctDNA-Analyse mittels NGS als Alternative. Daher empfehlen die Leitlinien der European Society for Medical Oncology (ESMO) den Nachweis somatischer Mutationen anhand von Gewebe- oder Plasmaproben und raten darüber hinaus insbesondere nach Progress unter CDK4/6i zur Testung von g*BRCA1/2* oder g*PALB2* [11]. In ähnlicher Weise empfiehlt die American Society of Clinical Oncology (ASCO) die Testung von Gewebe oder ctDNA auf Biomarker wie *PIK3CA-, BRCA1/2-* und *ESR1*-Mutationen [53, 54].

**Der Einsatz der Liquid Biopsy sollte somit sowohl beim erstmaligen Nachweis einer Metastasierung als auch bei jedem weiteren Progress in Erwägung gezogen werden – insbesondere, wenn molekulare Marker die Therapiewahl beeinflussen können.**

## Szenario: Patientin ist Kandidatin für ET ± zielgerichtete Therapie

### Optionen bei Fehlen molekularer Alterationen oder zielgerichteter Therapien

Wenn keine zielgerichtete Therapie verfügbar ist oder im Einzelfall keine therapierelevante Alteration vorliegt, können laut ESMO Metastatic Breast Cancer Living Guidelines (V1.2 April 2025) bei Patientinnen, bei denen keine Indikation zur Umstellung auf eine zytotoxische Therapie besteht und eine Fortführung der endokrin-basierten Behandlung weiterhin vertretbar erscheint, folgende Strategien erwogen werden: Kombination von Everolimus mit Exemestan, Everolimus mit Fulvestrant, ein Wechsel des endokrinen Partners ± CDK4/6i oder Fulvestrant-Monotherapie [11].

In der BOLERO-2-Studie zeigte Everolimus + Exemestan bei CDK4/6i-naiven Patientinnen Wirksamkeit [55, 56], doch Real-World-Daten belegen ein kürzeres behandlungsfreies Intervall nach vorheriger CDK4/6i-Therapie (4,3 Monate gegenüber 6,2 Monaten unter alleiniger ET in der Zweitlinie bzw. 4,1 Monate gegenüber 5,6 Monaten in der Drittlinie) [57]. Zudem traten therapiebedingte Abbrüche bei 19 % unter Everolimus vs. 4 % unter Placebo auf [55].

Mehrere Phase-II-Studien (MAINTAIN [58], PALMIRA [59], PACE [60]) untersuchten die CDK4/6i-Rechallenge mit teils widersprüchlichen Ergebnissen (Tab. 1). Nur in MAINTAIN bewirkte Ribociclib + Fulvestrant einen PFS-Vorteil gegenüber Fulvestrant allein, nicht jedoch bei Patientinnen mit *ESR1*-mutierten Tumoren. Hier ist jedoch zu beachten, dass die Mehrheit der Patientinnen in der MAINTAIN-Studie zuvor mit Palbociclib behandelt wurde (86,5 %). Im Gegensatz dazu, wurden Ribociclib und Abemaciclib – beides Substanzen, die heute aufgrund ihres Vorteils im Gesamtüberleben in der Erstlinie bevorzugt eingesetzt werden – nur bei 11,7 % bzw. 1,7 % der Patientinnen verabreicht [58]. Hier ist zu erwähnen, dass Ribociclib das mediane Gesamtüberleben um 12,5 Monate signifikant verlängerte (*p* = 0,008) [17], während Abemaciclib das mediane Gesamtüberleben um 13,1 Monate verlängerte und obwohl dieser Unterschied statistisch nicht signifikant war (*p* = 0,066), wird er aus klinischer Sicht als relevant eingeschätzt [18]. In PALMIRA und PACE zeigte Palbociclib keine zusätzliche Wirksamkeit [59, 60]. Ein Trend im Hinblick auf einen Zusatznutzen ergab sich in PACE durch die Kombination mit Avelumab (8,1 Monate; HR: 0,75 vs. Fulvestrant; *p* = 0,23) [60].

**Tab. 1:** Ergebnisse zur CDK4/6i-Rechallenge aus postMONARCH, MAINTAIN, PALMIRA und PACE

	postMONARCH [61]	MAINTAIN [58]	PALMIRA [59]	PACE [60]
*Phase*	3	2	2	2
*Patientinnen (n)*	368	119	198	220
*Chemotherapie-vorbehandelt*	0 %	9 %	0 %	16 %
*Palbociclib-vorbehandelt*	59 %	87 %	100 %	91 %
*Ribociclib-vorbehandelt*	33 %	12 %	0 %	5 %
*Abemaciclib-vorbehandelt*	8 %	2 %	0 %	4 %
*Interventionsarm*	**Abemaciclib**	**Ribociclib**	**Palbociclib**	**Palbociclib**
*Kontrollarm*	Fulvestrant	Fulvestrant oder Exemestan	Fulvestrant oder Letrozole	Fulvestrant
*mPFS in Monaten (CDKi* *+* *ET vs. ET)*	6,0 vs. 5,3	5,3 vs. 2,8	4,2 vs. 3,6	4,6 vs. 4,8
	HR: 0,73 (95 % CI: 0,57–0,95)	HR: 0,57 (95 % CI: 0,39–0,85)	HR: 0,80 (95 % CI: 0,6, 1,1)	HR: 1,11 (90 % CI: 0,79, 1,55)
	*p* = 0,02	*p* = 0,006	*p* = 0,206	*p* = 0,62
*mPFS ESR1-mut in Monaten (CDKi* *+* *ET vs. ET)*	Keine Angabe	3,0 (*n* = 18) vs. 3,0 (*n* = 15)	Keine Angabe	5,2 (*n* = 102) vs. 3,3 (*n* = 48)
		HR: 1,22 (95 % CI: 0,59–2,49)		HR: 0,68 (90 % CI: 0,42, 1,09)

Die Phase-III-Studie postMONARCH (Tab. 1) belegte erstmals einen signifikanten, wenn auch moderaten PFS-Vorteil für Abemaciclib + Fulvestrant im Vergleich zu Fulvestrant alleine nach CDK4/6i-Vortherapie (6,0 vs. 5,3 Monate; HR: 0,73). Der leichte Vorteil zeigte sich in allen Gruppen, auch bei Vorliegen von *ESR1*-, *AKT*- oder *PIK3CA*-Mutationen. Er wurde jedoch vor allem bei Patientinnen ohne viszerale Metastasen und mit vorheriger Palbociclib-Therapie sichtbar, wobei Subgruppenanalysen immer mit Vorsicht zu bewerten sind [61]. Die objektive Ansprechrate war mit Abemaciclib höher (17 % vs. 7 % bei messbarer Erkrankung). Primär scheinen allerdings die Patientinnen zu profitieren, die in der Erstlinie Palbociclib bekommen hatten [61]. Daten zum PFS bei Patientinnen mit Resistenzmutationen wurden noch nicht veröffentlicht. Basierend auf den Daten aus PALOMA‑3 [62] kann man erwarten, dass das PFS in beiden Gruppen (Abemaciclib + Fulvestrant sowie Placebo + Fulvestrant) kürzer ist.

**Die ESMO Metastatic Breast Cancer Living Guidelines (V1.2 April 2025) empfehlen daher, bei fehlenden therapierbaren Alterationen eine Kombination aus Fulvestrant und einem CDK4/6-Inhibitor-Switch (z.** **B. Abemaciclib oder Ribociclib) in Erwägung zu ziehen ([II, C; keine EMA-/FDA-Zulassungen]) oder alternativ eine Fulvestrant-Monotherapie ([I, C])** [11, 58, 61, 63]**. Ist die Patientin für eine mTOR-Inhibition geeignet, stellt Everolimus** **+** **Exemestan eine wirksame Option mit signifikant verlängertem PFS dar ([I, B])** [11, 55, 64]**. Auch Fulvestrant oder Tamoxifen können mit Everolimus kombiniert werden ([II, B]). Bei Einsatz von Everolimus ist eine Prophylaxe der Stomatitis mittels steroidalem Mundspülpräparat obligat ([V, A])** [11]**. Bei Vorliegen einer** ***ESR1*-Mutation (ESCAT I-A) kann, wenn Elacestrant nicht verfügbar ist oder bereits eingesetzt wurde, Fulvestrant** **+** **Everolimus bevorzugt werden ([V, B; keine EMA-/FDA-Zulassung])** [11, 65].

### *PI3K/AKT/PTEN*-alterierte Tumoren

Für Patientinnen mit HR+/HER2− mBC und *PIK3CA*-Mutationen stehen derzeit in Österreich der PI3Kα-selektive Inhibitor Alpelisib und der AKT-Inhibitor Capivasertib, jeweils in Kombination mit Fulvestrant, zur Verfügung. Die Zulassung von Alpelisib basiert auf der SOLAR-1-Studie, in der Alpelisib bei Patientinnen mit *PIK3CA*-mutierten Tumoren einen PFS-Vorteil bedingte (11,0 vs. 5,7 Monate; HR: 0,65; 95 % CI: 0,50–0,85; *p* < 0,001). In dieser Studie hatten jedoch nur wenige Patientinnen zuvor eine CDK4/6i-Therapie erhalten, weshalb die EMA-Zulassung derzeit auf die Kombination nach alleiniger endokriner Vorbehandlung beschränkt ist [66]. Die Phase-II-Studie BYlieve wurde initiiert, um die Sicherheit und Wirksamkeit von Alpelisib nach CDK4/6i-Therapie in einer breiteren Patientenkohorte zu untersuchen. In Kohorte A (nach CDK4/6i) lag das mediane PFS bei 7,3 Monaten (95 % CI: 5,6–8,3), mit einer 6‑Monats-PFS-Rate von 50,4 % (95 % CI: 41,2–59,6) – vergleichbar mit den Daten aus der SOLAR-1-Studie [67, 68, 69].

Capivasertib ist ein Inhibitor aller drei AKT-Isoformen und ist für HR+/HER2− mBC-Patientinnen mit *PIK3CA-, AKT1*- oder *PTEN*-Alterationen nach Progress unter ET zugelassen. In der CAPItello-291-Studie zeigte Capivasertib + Fulvestrant bei Patientinnen mit AKT-Pathway-veränderten Tumoren (davon 70 % mit vorheriger CDK4/6i-Therapie) gegenüber der Fulvestrant-Monotherapie einen PFS-Vorteil von 7,3 vs. 3,1 Monaten (HR: 0,50; 95 % CI: 0,38–0,65; *p* < 0,001) [70, 71]. Laut Subgruppenanalyse der CAPItello-291-Studie brachte Capivasertib + Fulvestrant auch bei CDK4/6i-vorbehandelten Patientinnen einen PFS-Vorteil mit sich (medianes PFS: 5,5 vs. 2,0 Monate), wenngleich dieser weniger ausgeprägt war als bei CDK4/6i-naiven Patientinnen (11,0 vs. 7,4 Monate) [72]. Wenn man nun einen Cross-Trial-Vergleich durchführt, zeigt sich, dass Therapieabbrüche aufgrund von Nebenwirkungen unter Capivasertib seltener waren als unter Alpelisib (13,0 % vs. 25,0 %). Auch hinsichtlich der Hyperglykämien fällt der Unterschied deutlich aus: während unter Alpelisib bei 36,6 % der Patient:innen eine Hyperglykämie auftrat (vs. 0,7 % in der jeweiligen Placebogruppe), waren es unter Capivasertib lediglich 2,3 % (vs. 0,3 %). Diarrhö war unter Capivasertib in der CAPItello-291-Studie häufiger (72,4 %) als unter Alpelisib in SOLAR‑1 (57,7 %). Die Häufigkeit kutaner Nebenwirkungen wie Hautausschlag war jedoch vergleichbar (38,0 % vs. 35,6 %) [70, 73].

**Die ESMO Metastatic Breast Cancer Living Guidelines (V1.2 April 2025) empfehlen bei Patientinnen mit Tumoren, die eine oder mehrere** ***PIK3CA*-,** ***AKT1*- oder** ***PTEN*-Alterationen aufweisen (ESCAT-Score: I/II), nach Progress unter mindestens einer endokrin basierten Therapie im metastasierten Stadium oder bei Rezidiv innerhalb von 12 Monaten nach Abschluss einer adjuvanten ET die Kombination aus Fulvestrant und Capivasertib (ESMO-MCBS v1.1 Score: 3; Evidenzstufe I, Empfehlung A) als Behandlungsoption** [11, 71].

**Für Patientinnen mit** ***PIK3CA*-mutierten Tumoren (Exon 7, 9 oder 20; ESCAT-Score: I‑A) nach Vorbehandlung mit einem AI mit oder ohne CDK4/6i und bei adäquatem HbA1c-Wert kann Fulvestrant in Kombination mit Alpelisib erwogen werden (ESMO-MCBS v1.1 Score: 2; Evidenzstufe I, Empfehlung B)** [11, 66, 73].

Ein weiterer PI3Kα-selektiver Inhibitor ist Inavolisib. Die Kombination Inavolisib, Palbociclib und Fulvestrant wurde bereits im Oktober 2024 von der FDA für Patientinnen mit *PIK3CA*-mutiertem, endokrinresistentem HR+/HER2− mBC zugelassen, bei denen ein Fernrezidiv während oder innerhalb von 12 Monaten nach Abschluss der adjuvanten endokrinen Therapie auftrat. In der INAVO120-Studie zeigte diese Triplet-Therapie ein medianes PFS von 17,2 Monaten gegenüber 7,3 Monaten im Vergleichsarm (HR: 0,42; 95 % CI: 0,32–0,55; Data cut-off, 15. November 2024). Inavolisib ist zudem der erste PIK3CA-Inhibitor, der einen statistisch signifikanten OS-Vorteil mit einer absoluten Differenz von 7 Monaten zeigt (34 vs 27 Monate; HR: 0,67; 95 % CI: 0,48–0,94; *p* < 0,0190). Zu den relevanten Nebenwirkungen von Inavolisib zählen Hyperglykämie, Stomatitis, Diarrhö und Hautausschlag. Dennoch blieb die Lebensqualität erhalten, und die Therapieabbruchrate belief sich lediglich auf 6,2 % (vs. 0,6 % im Kontrollarm) [74, 75, 76].

**Abb. 2:**
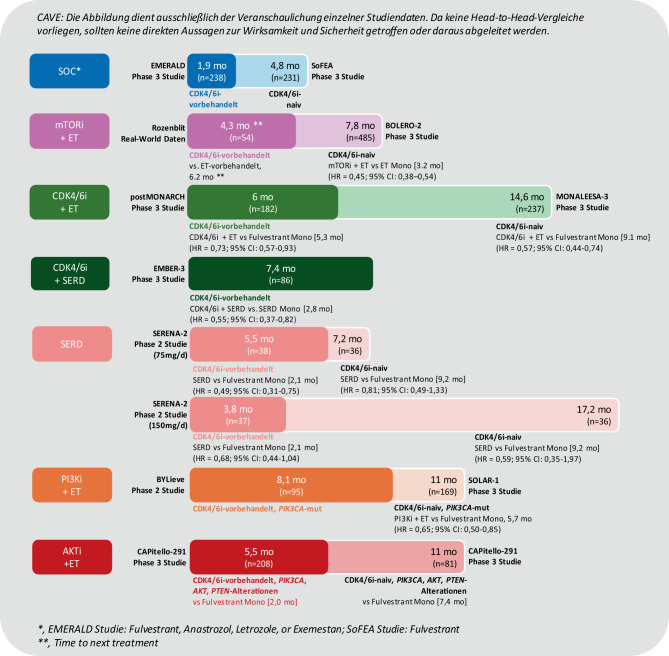
Wirksamkeit von 2L+ ET-basierten Therapien bei HR+/HER2− mBC mit oder ohne vorangegangene CDK4/6i-Therapie. EMERALD [77]; SoFEA [78]; Rozenblit [57]; BOLERO‑2 [79]; postMONARCH [80]; MONALEESA‑3 [81]; EMBER‑3 [82], SERENA‑2 [83]; BYLieve [84]; SOLAR‑1 [73], CAPitello-291 [72]

Die Wirksamkeit einzelner 2L+ ET-basierter Therapien bei HR+/HER2− mBC mit oder ohne vorangegangene CDK4/6i-Therapie ist in Abb. 2 zusammengefasst.

### *ESR1* als therapeutisches Target

Während die bisherigen Studien zur CDK4/6i-Fortsetzung oder -Rechallenge teils widersprüchliche Ergebnisse lieferten und der Nutzen zumeist moderat bleibt, rückt nun ein neues Therapiekonzept in den Fokus: die selektiven Östrogenrezeptor-Degrader (SERDs).

Elacestrant, der erste zugelassene orale SERD, stellt eine Therapieoption für Patientinnen mit *ESR1*-Mutationen nach Fortschreiten unter CDK4/6-Inhibitoren dar. Die Zulassung basiert auf der Phase-III-Studie EMERALD, in der Elacestrant im Vergleich zur endokrinen Standardtherapie bei Patientinnen mit HR+/HER2− mBC ein signifikant längeres PFS zeigte (HR: 0,70; 95 % CI: 0,55–0,88; *p* = 0,002). Besonders deutlich war der Nutzen bei Patientinnen mit *ESR1*-mutierten Tumoren (HR: 0,55; 95 % CI: 0,39–0,77; *p* = 0,0005), mit einer 12-Monats-PFS-Rate von 26,8 % vs. 8,2 % im Vergleichsarm [77]. Eine Post-Hoc-Analyse ergab, dass Patientinnen mit *ESR1*-Mutation und einer CDK4/6i-Vortherapie von mindestens 12 Monaten ein medianes PFS von 8,6 Monaten unter Elacestrant gegenüber 1,9 Monaten unter Standardtherapie erreichten. Bei kürzerer Vorbehandlung (≥ 6 Monate) lag das PFS bei 4,1 vs. 1,9 Monaten [85]. Bei Vorliegen kombinierter *ESR1*- und *PIK3CA*-Mutationen war das mediane PFS unter Elacestrant zwar ebenfalls verbessert, aber numerisch geringer (5,5 vs. 1,9 Monate) [86, 87].

Auffällig war der steile Abfall der Kaplan-Meier-Kurven bei allen Patientinnen – einschließlich der *ESR1*-mutierten Patientinnen – innerhalb der ersten zwei Monate, gefolgt von einer Separation der Kurven zugunsten von Elacestrant [86, 87]. Dieses Muster deutet darauf hin, dass ein relevanter Anteil der Patientinnen, unabhängig vom molekularen Subtyp, eine primäre endokrine Resistenz aufwies und somit nicht von einer endokrinen Monotherapie profitierte.

Ein ähnlicher PFS-Verlauf wurde auch bei einem weiteren oralen SERD beobachtet: Imlunestrant, ein Wirkstoff in klinischer Entwicklung, der aktuell in der Phase-III-Studie EMBER‑3 bei Patientinnen mit ER+/HER2− mBC nach Progress unter AI mit oder ohne CDK4/6i mit einer endokrinen Standardtherapie (Fulvestrant oder Exemestan) verglichen wird. In der Subgruppe der Patientinnen mit *ESR1*-Mutationen zeigte sich ein signifikanter Vorteil für Imlunestrant mit einem medianen PFS von 5,5 vs. 3,8 Monaten (HR: 0,62; 95 % CI: 0,46–0,82; *p* < 0,001). In der Gesamtpopulation war der Unterschied dagegen nicht signifikant (medianes PFS: 5,6 vs. 5,5 Monate; HR: 0,87; 95 % CI: 0,72–1,04; *p* = 0,12). Deutlich wirksamer zeigte sich hingegen die Kombination von Imlunestrant + Abemaciclib in der Gesamtpopulation, die ein medianes PFS von 9,4 Monaten gegenüber 5,5 Monaten unter Imlunestrant-Monotherapie erreichte (HR: 0,57; 95 % CI: 0,44–0,73; *p* < 0,001), unabhängig vom *ESR1*-Mutationsstatus. Bei Patientinnen mit einer *ESR1*-Mutation betrug das mediane PFS 11,1 Monate versus 5,5 Monate (HR: 0,53; 95 %-CI: 0,35–0,80), bei Patientinnen ohne *ESR1*-Mutation 9,1 Monate versus 5,5 Monate (HR: 0,59; 95 %-CI: 0,43–0,81). In einer beim ESMO Breast Congress 2025 präsentierten Subgruppenanalyse bestätigte sich der klare Vorteil für die Kombination Imlunestrant + Abemaciclib auch bei CDK4/6i-vorbehandelten Patientinnen, und zwar wiederum unabhängig vom *ESR1*-Mutationsstatus. Bei Patientinnen mit *ESR1*-Mutation betrug das mediane PFS 11,1 vs. 5,4 Monate (HR: 0,44; 95 % CI: 0,28–0,70), bei Patientinnen ohne *ESR1*-Mutation lag es bei 7,4 vs. 2,8 Monaten (HR: 0,55; 95 % CI: 0,37–0,82) [88]. Schwerwiegende Nebenwirkungen (Grad ≥ 3) traten bei 17,1 % unter Imlunestrant, 20,7 % unter Standardtherapie und 48,6 % unter der Kombinationstherapie auf [89]. EMBER‑3 ist somit die erste Phase-III-Studie, die einen Nutzen einer Kombination aus oralem SERD und CDK4/6i nach Krankheitsprogress unter vorheriger CDK4/6i-Therapie belegt [88]. Da der Nutzen in der Kombinationsgruppe unabhängig vom *ESR1*-Mutationsstatus war und es keinen Vergleich mit Abemaciclib-Monotherapie gibt, lässt sich derzeit allerdings noch nicht eindeutig feststellen, ob das Therapieansprechen hauptsächlich auf die Zugabe von Imlunestrant oder auf Abemaciclib selbst zurückzuführen ist. Eine Zulassung durch FDA oder EMA liegt derzeit noch nicht vor.

Camizestrant ist ein weiterer, oraler SERD und reiner ER-Antagonist, der in der Phase-II-Studie SERENA‑2 bei Patientinnen mit HR+/HER2− mBC nach endokriner Vortherapie – davon ca. 50 % CDK4/6i-vorbehandelt – ein signifikant längeres PFS im Vergleich zu Fulvestrant zeigte (medianes PFS: 7,2 Monate unter Camizestrant 75 mg bzw. 7,7 Monate unter Camizestrant 150 mg vs. 3,7 Monate; HR: 0,59; *p* = 0,017 bzw. HR: 0,64; *p* = 0,009). Der Vorteil bestätigte sich sowohl in der CDK4/6i-vorbehandelten Subgruppe (medianes PFS: 5,5 bzw. 3,8 vs. 2,1 Monate; HR: 0,49 bzw. 0,68), als auch bei *ESR1*-mutierten Tumoren (mPFS: 6,3 Monaten unter Camizestrant 75 mg bzw. 9,2 Monaten unter Camizestrant 150 mg vs. 2,2 Monaten mit Fulvestrant) [90]. Darüber hinaus, war eine frühe und anhaltende Reduktion der *ESR1*-mutierten ctDNA unter Camizestrant mit einem besseren PFS assoziiert [91]. In Analogie zu PADA‑1, untersuchte die Phase-III-Studie SERENA‑6 ein ctDNA-gesteuertes Therapiekonzept mit frühzeitigem Wechsel auf Camizestrant bei neu auftretender *ESR1*-Mutation unter AI + CDK4/6i noch vor der klinischen Progression [92]. Dabei zeigte sich ein statistisch signifikanter und klinisch relevanter PFS-Vorteil zugunsten der Camizestrant-Kombination [93]. Die Interpretation dieser Daten sollte zum jetzigen Zeitpunkt jedoch noch kritisch erfolgen. Zum einen bleibt unklar, ob es sich bei diesem Vorgehen um eine Modifikation der Erstlinientherapie oder eher um eine vorgezogene Zweitlinienstrategie handelt. Zum anderen wurde im Studiendesign kein Crossover vorgesehen, was die Validierung der zugrunde liegenden Hypothese – nämlich, dass ein frühzeitige Intervention vor klinischer Progression tatsächlich vorteilhaft ist – erschwert. Darüber hinaus stellen sich praktische Fragen zur Umsetzung eines solchen Konzepts: die Belastung durch regelmäßige ctDNA-Kontrollen, die psychologische Belastung durch das Warten auf molekulare Ergebnisse und die Auswirkungen auf das Gesundheitssystem müssen sorgfältig berücksichtigt werden [93]. Trotz der wichtigen Impulse für die Weiterentwicklung personalisierter Strategien bei HR+/HER2− mBC hängt die Empfehlung dieser Strategie jedoch davon ab, ob sich neben einem verlängerten PFS auch ein Überlebensvorteil und ein klarer klinischer Nutzen über mehrere Therapielinien hinweg belegen lassen – sowie von der Machbarkeit im klinischen Alltag und einer angemessenen Kosten-Nutzen-Bilanz.

**Die ESMO Metastatic Breast Cancer Living Guidelines (V1.2 April 2025) empfehlen den Einsatz von Elacestrant (sofern verfügbar) bei Patientinnen mit HR-positivem,** ***HER2*-negativem,** ***ESR1*-mutiertem fortgeschrittenem oder metastasiertem Mammakarzinom nach Progress unter mindestens einer endokrinen Therapielinie (ESCAT-Score: I‑A; ESMO-MCBS v1.1 Score: 3; Evidenzstufe I, Empfehlung A)** [11, 77].

**Abb. 3:**
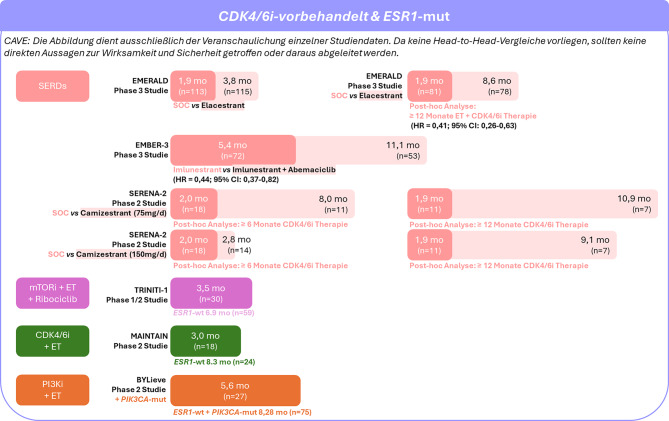
Wirksamkeit von 2L+ Therapien beim ESR1-mutierten HR+/HER2− mBC nach vorangegangener CDK4/6i-Therapie. EMERALD [77]; EMBER‑3 [82]; SERENA‑2 [83]; TRINITI‑1 [94]; MAINTAIN [58]; BYLieve [95]

Die Wirksamkeit einzelner 2L+ Therapien beim *ESR1*-mutierten HR+/HER2− mBC nach vorangegangener CDK4/6i-Therapie ist in Abb. 3 zusammengefasst.

### *BRCA/PALB2-*Keimbahnmutationen

Die meisten NGS-Panels beinhalten heute die Analyse von *BRCA*-Mutationen, wobei in der Regel die Allelfrequenz Rückschlüsse auf den Ursprung der Mutation (Keimbahn vs. somatisch) erlaubt [41]. In einer Metaanalyse von sieben Studien fanden sich Keimbahnmutationen in *BRCA1* bzw. *BRCA2* bei 17 % bzw. 41 % der Patientinnen mit HR-positivem Mammakarzinom [96]. Bei Patientinnen mit pathogenen Keimbahnmutationen in *BRCA1* oder *BRCA2* sollte eine Monotherapie mit einem PARP-Inhibitor (Olaparib oder Talazoparib) in Erwägung gezogen werden. Auch bei Keimbahnmutationen in *PALB2* oder bei somatischen *BRCA1/2*-Mutationen kann ein Einsatz diskutiert werden, wenngleich die Evidenzlage hier schwächer ist [11, 97]. Allerdings deuteten Daten der Phase-II-Studie TBRC 048 auf begrenzte Wirksamkeit von Olaparib bei ausschließlich somatischen *BRCA*-Mutationen hin [98].

**Die ESMO Metastatic Breast Cancer Living Guidelines (V1.2 April 2025) empfehlen die PARP-Inhibitor-Monotherapie mit Olaparib (ESMO-MCBS v1.1 Score: 4) oder Talazoparib (Score: 3) bei Patientinnen mit pathogenen Keimbahnmutationen in** ***BRCA1/2*** **(ESCAT-Score: I‑A; Evidenzstufe I, Empfehlung A) oder** ***PALB2*** **(ESCAT-Score: II‑B; Evidenzstufe I, Empfehlung A). Auch bei Tumoren mit somatischen pathogenen oder wahrscheinlich pathogenen** ***BRCA1/2*-Mutationen kann eine PARP-Inhibitor-Monotherapie in Erwägung gezogen werden (ESCAT-Score: II‑B; Evidenzstufe II, Empfehlung B)** [11].

## Szenario: Patientin ist keine Kandidatin für ET ± zielgerichtete Therapie

### HER2-low- & HER2-ultra-low-Tumoren sowie HER2-Negativität bei Hochrisikoerkrankung

Die Studien DESTINY-Breast04 und DESTINY-Breast06 (siehe Tab. 2) untersuchten das Antikörper-Wirkstoffkonjugat (ADC) Trastuzumab Deruxtecan (T-DXd) im Vergleich zur von den behandelnden Ärzt:innen gewählten Chemotherapie bei Patientinnen mit HER2-low- bzw. HER2-ultra-low-Tumoren [99, 100, 101].

**Tab. 2:** Antibody-drug-conjugates (*ADCs*) bei mBC

	Destiny-Breast-06 bei HR+/HER2-low und HER2-ultralow [99, 102]	Destiny-Breast-04 HR+/HER2-low [100, 101]	TROPION-B01 HR+/HER2− [103]	TROPICS-02 HR+/HER2− [104]
*Patientinnen (n)*	866	557	732	272
*Chemotherapie-vorbehandelt*	0	1–2	1–2	2–4
*CDK4/6i-vorbehandelt*	90,4 %	64,1 %	82 %	99 %
*ADC*	**T‑DXd**	**T‑DXd**	**Dato-DXd**	**SG**
*PFS (Monate)*	13,2 vs. 8,1	10,1 vs. 5,4	6,9 vs. 4,9	5,5 vs. 4,0
	HR: 0,62 (95 % CI: 0,52, 0,75)	HR: 0,51 (95 % CI: 0,40, 0,64)	HR: 0,63 (95 % CI: 0,52, 0,76)	HR: 0,66 (95 % CI: 0,53–0,83)
	*p* < 0,001	*p* < 0,0001	*p* < 0,0001	*p* = 0,0003
*OS*	HR: 0,83^§^ (95 % CI: 0,66, 1,05)	HR: 0,64 (95 % CI: 0,48, 0,86)	HR: 0,86 (95 % CI, 0,70, 1,06)	HR: 0,79 (95 % CI: 0,65–0,96)
	*p* = NR	*p* = 0,0028	*p* = NR	*p* = 0,02
*ORR (%)*	57,3 vs. 31,2	52,6 vs. 16,3	36,4 vs. 22,9	21,0 vs. 14,0

^§^data immature

Bei Patientinnen mit HER2-low mBC nach ein bis zwei vorangegangenen Chemotherapien verbesserte T‑DXd in der DESTINY-Breast04-Studie das mediane PFS (10,1 vs. 5,4 Monate; HR: 0,51; *p* < 0,0001) und Gesamtüberleben (OS; 23,9 vs. 17,5 Monate; HR: 0,64; *p* = 0,0028) unabhängig vom HER2-IHC-Status (1+ oder 2+). Grad-3-Nebenwirkungen traten unter T‑DXd seltener auf als unter ärztlich gewählter Chemotherapie (52,6 % vs. 67,4 %), jedoch kam es bei 12,1 % zu medikamentös assoziierten interstitiellen Lungenerkrankungen (ILD) oder Pneumonitiden, die in 0,8 % sogar tödlich verliefen [100, 101].

In der DESTINY-Breast06-Studie wurde T‑DXd bei chemotherapienaiven Patientinnen mit HR+/HER2-low (IHC 2+/ISH− oder IHC 1+) bzw. HR+/HER2-ultralow (IHC 0 mit Membranfärbung) mBC untersucht, die entweder ≥ 2 endokrinbasierte Therapien (± zielgerichtete Therapien) oder 1 ET erhalten hatten, sofern ein Progress ≤ 24 Monate nach adjuvanter ET bzw. ≤ 6 Monate nach ET + CDK4/6i auftrat – also primär endokrin resistente Erkrankungen. Das mediane PFS betrug in der mit T‑DXd behandelten HER2-low-Population 13,2 Monate gegenüber 8,1 Monaten im Chemotherapiearm (HR: 0,62; *p* < 0,001). Im ITT-Kollektiv (HER2-low + ultralow) betrug das mediane PFS 13,2 vs. 8,1 Monate (HR: 0,64; *p* < 0,001) [102]. Im Unterschied zur DESTINY-Breast04-Studie, in der kein signifikanter Unterschied zwischen IHC 1+ und 2+ bestand [100, 101], deutete DESTINY-Breast06 auf tendenzielle Unterschiede in Abhängigkeit vom HER2-Expressionsniveau hin, was die Relevanz einer präzisen Patientinnenselektion unterstreicht [99, 100, 101]. In einer Subgruppenanalyse profitierten nämlich Patientinnen mit IHC 2+/ISH− stärker von T‑DXd (medianes PFS: 15,2 vs. 7,0 Monate; HR: 0,43) als solche mit IHC 1+ (medianes PFS: 12,9 vs. 8,2 Monate; HR: 0,74) [102]. Obwohl die Zahl der HER2-ultralow-Patientinnen begrenzt war, zeigte sich auch in dieser Subgruppe ein statistisch signifikanter PFS-Benefit, welcher mit der von Patientinnen in der IHC 1+ Gruppe vergleichbar war (medianes PFS: 13,2 vs. 8,3 Monate; HR: 0,78) [99]. Bezüglich der Sicherheit fiel eine ILD-/Pneumonitisrate von 11,3 % auf. Im T‑DXd-Arm wurden drei Fälle mit Grad-3-Ereignissen und drei letal verlaufene Fälle dokumentiert, während im Chemotherapie-Arm nur eine Patientin eine ILD/Pneumonitis Grad 2 entwickelte [99].

**Wie im Dezember 2024 von österreichischen Expert:innen bereits im Therapiealgorithmus postuliert** [105]**, erhielt T‑DXd im April 2025 in der Europäischen Union die Zulassung als Monotherapie zur Behandlung von erwachsenen Patientinnen mit nicht resezierbarem oder metastasiertem, HR+/HER2-low (IHC 1+ oder IHC 2+/ISH−) oder HER2-ultralow (IHC 0 mit Membranfärbung) Mammakarzinom, die bereits mindestens eine ET im metastasierten Setting erhalten haben und als nicht geeignet für eine weitere ET angesehen werden.**

Ebenfalls zugelassen für HR+/HER2− mBC nach ET und ≥ 2 zusätzlichen systemischen Therapien ist Sacituzumab Govitecan (SG), ein Trop-2-gerichtetes ADC. Darüber hinaus besitzt SG auch die Zulassung für Patientinnen mit metastasiertem triple-negativem Mammakarzinom, die ≥ 2 Vortherapien erhalten haben (davon ≥ 1 im metastasierten Setting) [104, 106, 107]. In der Phase-III-Studie TROPICS-02 (Tab. 2) wurde SG bei HR+/HER2− mBC untersucht. Eingeschlossene Patientinnen nach Vorbehandlung mit mindestens einem Taxan, einem CDK4/6i sowie einer ET zeigten unter SG ein verbessertes medianes PFS (+1,5 Monate) und OS (+3,2 Monate) im Vergleich zur Chemotherapie. Häufigste unter Sacituzumab Govitecan (SG) aufgetretene unerwünschte Ereignisse (TEAEs) waren Neutropenie (71 % vs. 55 % unter Chemotherapie; Grad ≥ 3: 51 % vs. 39 %), Diarrhö (62 % vs. 23 %; Grad ≥ 3: 10 % vs. 1 %), Übelkeit (59 % vs. 35 %), Alopezie (48 % vs. 18 %) und Anämie (37 % vs. 28 %). Behandlungsbedingte Pneumonitis wurde unter SG nicht beobachtet (vs. zwei Fälle unter Chemotherapie, davon einer Grad ≥ 3). Therapieabbrüche infolge unerwünschter Ereignisse waren in beiden Armen niedrig (6 % vs. 4 %) [104].

Datopotamab Deruxtecan (Dato-DXd) ist das dritte als Monotherapie zur Behandlung von erwachsenen Patientinnen mit inoperablem oder metastasiertem HR+/HER2− Mammakarzinom zugelassene ADC. Die Indikation gilt nach Vorbehandlung mit einer ET und mindestens einer Chemotherapie im fortgeschrittenen Stadium. Dato-DXd setzt ebenso wie SG an Trop‑2 an und enthält denselben zytotoxischen Wirkstoff („Payload“) wie T‑DXd. In der TROPION-B01-Studie (Tab. 2) bewirkte Dato-DXd verglichen mit der Chemotherapie ein verlängertes medianes PFS (+2 Monate) und eine höhere objektive Responserate (36,4 % vs. 22,9 %). Häufigste TEAEs unter Dato-DXd waren Übelkeit (51 % vs. 24 %), Stomatitis (50 % vs. 13 %), Alopezie (36 % vs. 21 %) und Neutropenie (11 % vs. 43 %). Neben der Stomatitis ist eine weitere praxisrelevante Nebenwirkung die Augentoxizität. Okuläre Nebenwirkungen waren nämlich unter Dato-DXd häufiger (40 % vs. 12 %), dennoch in den meisten Fällen milder Ausprägung; nur drei Grad 3-Fälle (trockene Augen, punktförmige/ulzerative Keratitis) wurden berichtet. Dosisreduktionen (21 % vs. 30 %) und -unterbrechungen (12 % vs. 25 %), sowie schwerwiegende therapiebedingte unerwünschte Ereignisse (SAEs) (6 % vs. 9 %) und Therapieabbrüche (2,5 % vs. 2,6 %) waren unter Dato-DXd seltener. In Diskordanz zu T‑Dxd, therapiebedingte ILD/Pneumonitis wurde lediglich bei 3,3 % unter Dato-DXd beobachtet (meist Grad 1/2; zwei Grad 3, ein Grad 5) [103, 108]. Die OS-Daten, welche in der ESMO Virtual Plenary am 12. Februar 2025 von Barbara Pistilli vorgestellt wurden, zeigten im Gegensatz zu SG, trotz wenig vorbehandeltem Patientenkollektiv, keinen signifikanten Vorteil von Dato-DXd gegenüber der Chemotherapie (18,6 vs. 18, 3 Monate; HR: 1,01; 95 % CI: 0,83–1,22).

Die optimale Sequenzierung von ADCs bei Patientinnen mit mBC ist derzeit ein *unmet need* und Gegenstand laufender Diskussionen und klinischer Forschung. Zur Beantwortung dieser wichtigen, klinisch relevanten Frage sind weitere Erkenntnisse, auch aus dem Frühstadium [109] erforderlich. Künstliche Intelligenz-Modelle könnten uns in Zukunft auch bei der Beantwortung dieser Frage helfen, da bereits kollaborative Initiativen für maschinelles Lernen entwickelt wurden, die in der Lage sind, große Datenmengen mit verschiedenen Einrichtungen weltweit auszutauschen und dabei den Datenschutz zu wahren [110].

**Die ESMO Metastatic Breast Cancer Living Guidelines (V1.2 April 2025) empfehlen Trastuzumab Deruxtecan bei Patientinnen mit HER2-low oder HER2-ultralow mBC nach mindestens zwei endokrinen Therapielinien im metastasierten Setting oder nach einer ET-Linie im metastasierten Setting, wenn ein Progress innerhalb von 24 Monaten nach adjuvanter ET oder innerhalb von 6 Monaten unter ET mit einem CDK4/6i aufgetreten ist [Evidenzstufe I, Empfehlung B]** [11, 99, 102]. **Trastuzumab Deruxtecan [ESMO-MCBS v1.1 Score: 4] wird bei Patientinnen mit HER2-low mBC nach einer Chemotherapielinie empfohlen [Evidenzstufe I, Empfehlung A]** [100, 111]. **Sacituzumab Govitecan [ESMO-MCBS v1.1 Score: 4] sollte bei Patientinnen mit HR+/HER2− mBC nach mindestens zwei Chemotherapielinien in Betracht gezogen werden [Evidenzstufe I, Empfehlung B]** [11, 112]. **Datopotamab Deruxtecan kann bei Patientinnen mit HR+/HER2− mBC, die zuvor mit Chemotherapie behandelt wurden, in Erwägung gezogen werden [Evidenzstufe I, Empfehlung B]** [11, 102]. **Sacituzumab Govitecan kann bei Patientinnen mit HR+, HER2-low oder HER2-ultralow mBC nach Vorbehandlung mit Trastuzumab Deruxtecan berücksichtigt werden [Evidenzstufe V, Empfehlung B]** [11].

### Chemotherapie

Schließlich bleiben auch Chemotherapien ein wichtiger Bestandteil der Behandlung. Bevorzugt wird eine sequenzielle Monochemotherapie [II, A]; Kombinationstherapien sind bei drohendem Organversagen mit raschem Therapiebedarf zu erwägen [V, A]. Für die Einzelsubstanztherapie stehen u. a. Anthrazykline, Taxane, Capecitabin, Eribulin, Vinorelbin und platinhaltige Substanzen zur Auswahl. Eine Rechallenge mit Anthrazyklinen oder Taxanen ist bei einem krankheitsfreien Intervall ≥ 12 Monate nach adjuvanter Chemotherapie möglich; dabei können liposomale Anthrazykline oder nab-Paclitaxel eingesetzt werden. Die Chemotherapie sollte bis zur Progression oder inakzeptabler Toxizität fortgeführt werden – ausgenommen Anthrazykline, bei denen kumulative Dosisgrenzen zu beachten sind [II, B]. Eine optimale Sequenz zytotoxischer Substanzen ist bislang nicht etabliert; die Therapiewahl sollte individuell mit der Patientin abgestimmt werden. Die Kombination aus einem Taxan oder Capecitabin mit Bevacizumab [ESMO-MCBS v1.1 Score: 3; EMA-Zulassung, keine FDA-Zulassung] stellt – sofern verfügbar – eine Option in der Erstlinie dar [I, C] [11].

## Neue Medikamente am Horizont

PROTACs (PROteolysis TArgeting Chimera) sind kleine Moleküle, die den Östrogenrezeptor zur Degradation über das Ubiquitin-Proteasom-System anvisieren. Vepdegestrant ist ein neuer oraler PROTAC, dessen klinische Aktivität bereits in der Phase-1/2-Studie VERITAC nachgewiesen wurde. An dieser nahmen Patientinnen mit HR+/HER2− metastasiertem Brustkrebs (mBC) teil, die im Median vier vorangegangene Therapielinien – einschließlich CDK4/6-Inhibitoren – erhalten hatten. Die Phase-III-Studie VERITAC‑2, vorgestellt am ASCO 2025, verglich die Wirksamkeit und Sicherheit von Vepdegestrant mit Fulvestrant bei Patientinnen mit HR+/HER2− mBC nach endokriner Therapie mit CDK4/6i. Bei Patientinnen mit *ESR1*-mutierten Tumoren, führte Vepdegestrant im Vergleich zu Fulvestrant zu einer Verdoppelung des mPFS (5,0 vs. 2,1 Monate; HR: 0,57; CI 95 % 0,42–0,77; *p* = 0,0001). In der Gesamtpopulation wurde hingegen kein signifikanter Unterschied festgestellt. Zu den häufigsten Nebenwirkungen von Vepdegestrant zählten: Fatigue, Transaminase-Erhöhungen und Übelkeit. Dennoch war die Therapieabbruchsrate in der Vepdegestrant-Gruppe mit 2,9 % sehr niedrig (vs 0,7 % in der Fulvestrant-Gruppe). Erwähnenswert ist, dass in Analogie zu den oralen SERDs Elacestrant und Imlunestrant auch der PROTAC Vepdegestrant einen initalen steilen PFS-Abfall in der VERITAC‑2 Studie zeigt [113]. Diese Daten unterstreichen somit die Notwendigkeit weiterer Forschung, um Patientinnen mit limitierter endokriner Sensitivität besser identifizieren zu können und die Behandlungsstrategien gezielter anpassen zu können. Die Zulassung von Vepdegestrant wird derzeit von der FDA geprüft und könnte das therapeutische Armamentarium für Patientinnen mit *ESR1*-mutiertem HR+/HER2− mBC erweitern [114, 115, 116].

Lasoxifen ist ein selektiver Östrogenrezeptor-Modulator (SERM), der bereits in der Phase-II-Studie ELAINE 1 im Vergleich zu Fulvestrant bei Patientinnen mit *ESR1*-mutiertem HR+/HER2− mBC nach CDK4/6i-Vortherapie ein längeres mPFS zeigte (6,04 vs 4,04 Monate; HR 0,699; CI 95 % 0,445–1,125; *p* = 0,138) [117]. In der laufenden Phase-III-Studie ELAINE 3 wird die Kombination von Lasoxifen + Abemaciclib gegenüber Fulvestrant + Abemaciclib untersucht [118].

Auch selektive Androgenrezeptor-Modulatoren (SARM) stellen eine neue endokrine Behandlungsoption dar. Der orale SARM Enobosarm zeigte in einer Phase-II-Studie vielversprechende Ergebnisse (mPFS 5,6 Monate in der 9 mg-Gruppe und 4,2 Monate in der 18 mg-Gruppe). Allerdings war nur ein geringer Anteil der Patientinnen mit zielgerichteten Therapien, darunter CDK4/6-Inhibitoren, vorbehandelt. Die Wirksamkeit der Kombination Enobosarm + Abemaciclib nach Palbociclib-Vortherapie bei Patientinnen mit Androgenrezeptor-positivem, HR+/HER2− mBC wird derzeit in der Phase-III-Studie ENABLAR‑2 geprüft [119].

## Fazit für die Praxis

Die Therapie des HR+/HER2− mBC nach Progress unter CDK4/6i-Therapie erfordert ein individuelles, biomarkerbasiertes Vorgehen, um das Fortschreiten der Erkrankung zu verzögern und die Lebensqualität zu erhalten.

- Biomarker-Testungen mittels Gewebe und ctDNA sollten sowohl initial als auch bei Progress erfolgen, um therapierbare Alterationen wie *ESR1-, PIK3CA-, AKT1-, PTEN*- oder *BRCA1/2*-Mutationen zu identifizieren [11, 30, 31, 32, 33, 34, 35, 36, 37, 38, 47, 65, 66, 67, 70, 71, 74, 77, 120].
- Zielgerichtete Kombinationstherapien wie Fulvestrant + Capivasertib (CAPItello-291) [70, 71] oder Fulvestrant + Alpelisib (SOLAR‑1, BYlieve) [66, 67, 68, 69] verlängern das PFS bei *PI3K/AKT/PTEN*-Alterationen signifikant.
- Bei *ESR1*-Mutationen ist Elacestrant eine effektive Option (EMERALD-Studie) [77, 85, 86, 87]; kombinierte Ansätze wie Imlunestrant + Abemaciclib (EMBER-3) [88, 89] oder Camizestrant zeigen ebenfalls vielversprechende Ergebnisse [52, 90].
- Antikörper-Wirkstoffkonjugate (ADCs) wie Trastuzumab Deruxtecan (T-DXd), Sacituzumab Govitecan (SG) oder Datopotamab Deruxtecan (Dato-DXd) sind wirksame Optionen bei endokriner Resistenz, insbesondere T‑DXd bei HER2-low- oder HER2-ultralow-Tumoren [99, 100, 101, 102, 103, 104, 108].
- Chemotherapie bleibt ein zentraler Bestandteil im fortgeschrittenen Therapieverlauf – bevorzugt sequenziell als Monotherapie, ggf. in Kombination mit Bevacizumab [11].
- Die therapeutische Entscheidung sollte ganzheitlich getroffen werden – unter Berücksichtigung von Bildgebung (immer im Vergleich zum Ausgangsbefund), klinischem Verlauf, Tumormarkern und Patient:innenpräferenz [11, 121, 122].
- Begleiterkrankungen (z. B. kardiovaskuläre Erkrankungen, Diabetes, Autoimmunerkrankungen) erfordern eine individualisierte Therapiewahl. Zur Bewertung von Wirksamkeit, Toxizität und Lebensqualität kann die ESMO-Magnitude of Clinical Benefit Scale (ESMO-MCBS) herangezogen werden – ein validiertes Instrument zur objektiven Einschätzung des klinischen Nutzens onkologischer Therapien [122].

**Abb. 4:**
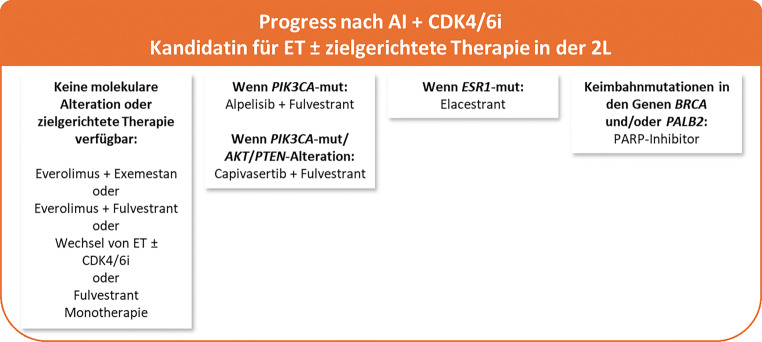
Kandidatin für ET ± zielgerichtete Therapie in der 2L nach Progress nach AI + CDK4/6i laut ESMO Metastatic Breast Cancer Living Guidelines (V1.2, April 2025) [11]

**Der in diesem DFP-Literaturstudium vorgestellte Therapiealgorithmus der ESMO Metastatic Breast Cancer Living Guidelines (V1.2, April 2025; siehe** Abb. 4 und 5**) wird sich durch die Zulassung der beschriebenen neuen Substanzen in Zukunft weiterentwickeln.**

**Abb. 5:**
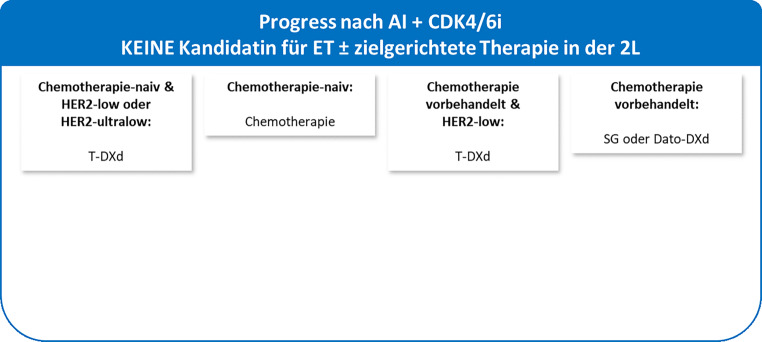
Keine Kandidatin für ET ± zielgerichtete Therapie in der 2L nach Progress nach AI + CDK4/6i laut ESMO Metastatic Breast Cancer Living Guidelines (V1.2, April 2025) [11]

## DFP-Fragen

Die primäre endokrine Resistenz wird klinisch definiert durch? (1 richtige Antwort)

Progress innerhalb der ersten 6 Monate einer endokrinen Erstlinientherapie, unabhängig von einer CDK4/6i-Therapie

Progress nach ≥ 6 Monaten einer endokrinen Erstlinientherapie oder Progress unter ET ab der Zweitlinie

Alterationen im PI3K/AKT/mTOR-Signalweg

*ESR1*-Mutationen die nach vorheriger endokriner Therapie bei mBC auftreten

Welche Aussagen zur Liquid Biopsy sind korrekt? (2 richtige Antworten)

Ersetzt die Gewebebiopsie vollständig

Ermöglicht serielles molekulares Monitoring

Kann nicht für *ESR1*-Mutationsanalyse verwendet werden

Ist die bevorzugte Methode zum Nachweis einer *ESR1*-Mutation

Welche Aussage zu HR+/HER2− metastasierten Mammakarzinom mit *ESR1* Mutation treffen zu? (2 richtige Antworten)

*ESR1*-Mutationen sind ein häufiges Merkmal der erworbenen Resistenz, typischerweise nach vorheriger endokriner Therapie

*ESR1*-Mutationen sind mit einer erhöhten Wirksamkeit von Aromatasehemmern assoziiert

Orale SERDs stellen bei *ESR1*-mutierten Tumoren eine effektive Therapieoption dar

*ESR1*-Mutationen lassen sich ausschließlich in Gewebeproben nachweisen

Welche *PIK3CA*-zielgerichteten Therapiekombination sind lt. Stand Juni 2025 in Europa zugelassen? (2 richtige Antworten)

Alpelisib + AI

Alpelisib + Fulvestrant

Capivasertib + Fulvestrant

Capivasertib + Abemaciclib

Welche Nebenwirkung trat unter Capivasertib in der CAPItello-291-Studie häufiger auf als unter Alpelisib in der SOLAR‑1 Studie? (1 richtige Antwort)

Hyperglykämie

Diarrhö

Hautausschlag

Die oben genannten Nebenwirkungen traten im Cross-Trial-Vergleich ähnlich oft auf

Welche Aussagen zu Inavolisib sind korrekt? (2 richtige Antworten)

Die INAVO120-Studie zeigte keinen PFS-Vorteil für die Triplet-Kombination bestehend aus Inavolisib, Palbociclib und Fulvestrant

Inavolisib ist ein PI3Kα-selektiver Inhibitor

Inavolisib ist zudem der erste PIK3CA-Inhibitor, der einen statistisch signifikanten OS-Vorteil mit einer absoluten Differenz von 7 Monaten zeigt

Inavolisib ist ein ESR1-Inhibitor

Welche Aussage trifft auf Elacestrant, Imlunestrant und Camizestrant zu? (1 richtige Antwort)

Es handelt sich um mTOR-Inhibitoren

Sie gehören zur Klasse der selektiven Estrogenrezeptor-Modulatoren (SERM)

Es handelt sich um orale selektive Estrogenrezeptor-Degrader (SERDs)

Sie sind CDK4/6-Inhibitoren der zweiten Generation

Welche Substanz bzw. Kombination erzielte in EMBER‑3 das beste PFS? (1 richtige Antwort)

Imlunestrant-Monotherapie

Abemaciclib-Monotherapie

Imlunestrant + Abemaciclib

Fulvestrant + Exemestan

Welche ADCs sind mit Stand Juni 2025 beim HR+/HER2− mBC zugelassen? (3 richtige Antworten)

Trastuzumab Deruxtecan

Sacituzumab Govitecan

Datopotamab Deruxtecan

Puxitatug Samrotecan

Welche Faktoren sollten bei der Wahl der Therapie beim HR+/HER2− metastasierten Mammakarzinom berücksichtigt werden? (3 richtige Antworten)

Wirksamkeit, Toxizitätsprofil und Lebensqualität

Ganzheitliche Entscheidungsfindung (Erkrankungsbiologie, Symptomatik, klinischer Verlauf, und Patient:innenpräferenz)

Individualisierte Therapiewahl unter Berücksichtigung von Begleiterkrankungen

Molekulare Alterationen sollten als einziges Entscheidungskriterium herangezogen werden

Welche Aussagen zum Einsatz von PARP-Inhibitoren bei HR+/HER2− metastasiertem Mammakarzinom treffen laut ESMO-Leitlinien (Stand April 2025) zu? (2 richtige Antworten)

Eine PARP-Inhibitor-Monotherapie mit Olaparib oder Talazoparib sollte bei pathogenen Keimbahnmutationen in *BRCA1/2* in Erwägung gezogen werden

Eine PARP-Inhibitor-Therapie ist ausschließlich bei somatischen *BRCA1/2*-Mutationen zugelassen

Auch bei Keimbahnmutationen in PALB2 kann eine PARP-Inhibitor-Therapie erwogen werden

Eine *BRCA1/2*-Mutation lässt sich nur mittels Gewebebiopsie und nicht über Allelfrequenzanalyse im NGS sicher zuordnen

Welche Aussagen zur Wirkung von Trastuzumab Deruxtecan (T-DXd) bei HR+/HER2− metastasiertem Brustkrebs treffen zu? (2 richtige Antworten)

T‑DXd zeigte in Studien einen PFS-Vorteil gegenüber Chemotherapie sowohl bei HER2-low als auch bei HER2-ultralow Tumoren

Die Wirksamkeit von T‑DXd ist bei HER2-ultralow-Tumoren im Vergleich zu HER2-IHC 2+ deutlich stärker ausgeprägt

Pneumonitis/Interstitielle Lungenerkrankungen gehören zu den häufigsten schwerwiegenden Nebenwirkungen unter T‑DXd

T‑DXd wirkt nur bei HER2-amplifizierten Tumoren mit ISH-positivem Nachweis

## Interessenkonflikt

V. Castagnaviz: Speaking Honoraria: Daiichi Sankyo, Astra Zeneca, Novartis, Roche. Adivisory board/Consulting Honoraria: Novartis, Roche, Pfizer. Travel and conference attendance: Daiichi Sankyo, Astra Zeneca, Gilead, Pierre-Fabre, Eli-Lilly, Menarini-Stemline, Roche, Novartis, Pfizer. M. Gnant: Personal fees/travel support from Amgen, AstraZeneca, Bayer, DaiichiSankyo, EliLilly, EPG Health (IQVIA), Menarini-Stemline, MSD, Novartis, PierreFabre, Veracyte. R. Bartsch: Advisory Role: Astra-Zeneca, Daiichi, Eisai, Eli-Lilly, Gilead, Gruenenthal, MSD, Novartis, Pfizer, Pierre-Fabre, Roche, Seagen, Stemline. Lecture Honoraria: Astra-Zeneca, BMS, Daichi, Eisai, Eli-Lilly, Gilead, Gruenenthal, MSD, Novartis, Pfizer, Pierre-Fabre, Roche, Seagen, Stemline. Research Support: Daiichi, MSD, Novartis, Roche. A. Fenzl gibt an, dass kein Interessenkonflikt besteht.
