# Correction: Was kommt nach CDK4/6-Inhibition? Perspektiven beim fortgeschrittenen Mammakarzinom

**DOI:** 10.1007/s00508-025-02622-7

**Published:** 2025-09-22

**Authors:** Vanessa Castagnaviz, Anna Fenzl, Michael Gnant, Rupert Bartsch

**Affiliations:** 1 Department of Internal Medicine III with Hematology, Medical Oncology, Hemostaseology, Infectiology and Rheumatology, Oncologic Center, Salzburg Cancer Research Institute – Laboratory for Immunological and Molecular Cancer Research (SCRI-LIMCR), Paracelsus Medical University Salzburg, Salzburg, Österreich; 2 Springer-Verlag GmbH, Wien, Österreich; 3 Comprehensive Cancer Center, Austrian Breast and Colorectal Cancer Study Group, Medical University of Vienna, Waehringer Guertel 18–20, 1090 Wien, Österreich; 4 Department of Medicine I, Division of Oncology, Medical University of Vienna, Wien, Österreich; 5 Landeskrankenhaus Salzburg, Müllner Hauptstraße, 5020 Salzburg, Österreich

**Correction:**

**Wien Klin Wochenschr 2025**

10.1007/s00508-025-02582-y

In diesem Artikel fehlte am Ende das Formular zur Teilnahme an der DFP-Fortbildung. Dieses wurde nun ergänzt.

Der Originalbeitrag wurde korrigiert.

